# Liberalized family presence policies in the intensive care unit: strategies for successful implementation

**DOI:** 10.62675/2965-2774.20250094

**Published:** 2025-09-11

**Authors:** Regis Goulart Rosa, Maria Clara Formolo de Souza, Pedro d´Elia Machado Silva, Rafael Barberena Moraes, Cassiano Teixeira

**Affiliations:** 1 Hospital Moinhos de Vento Department of Internal Medicine Porto Alegre RS Brazil Department of Internal Medicine, Hospital Moinhos de Vento - Porto Alegre (RS), Brazil.; 2 Universidade Federal do Rio Grande do Sul Faculty of Medicine Department of Intensive Medicine Porto Alegre RS Brazil Department of Intensive Medicine, Faculty of Medicine, Universidade Federal do Rio Grande do Sul - Porto Alegre (RS), Brazil.; 3 Universidade Federal do Rio Grande do Sul Postgraduate Program in Pneumological Sciences Porto Alegre RS Brazil Postgraduate Program in Pneumological Sciences, Universidade Federal do Rio Grande do Sul - Porto Alegre (RS), Brazil.; 4 Universidade Federal do Rio Grande do Sul Hospital de Clínicas de Porto Alegre Porto Alegre RS Brazil Hospital de Clínicas de Porto Alegre, Universidade Federal do Rio Grande do Sul - Porto Alegre (RS), Brazil.; 5 Universidade de Ciências da Saúde de Porto Alegre Department of Internal Medicine and Rehabilitation Sciences Porto Alegre RS Brazil Department of Internal Medicine and Rehabilitation Sciences, Universidade de Ciências da Saúde de Porto Alegre - Porto Alegre (RS), Brazil.; 6 Universidade Federal do Rio Grande do Sul Hospital de Clínicas de Porto Alegre Department of Critical Care Porto Alegre RS Brazil Department of Critical Care, Hospital de Clínicas de Porto Alegre, Universidade Federal do Rio Grande do Sul - Porto Alegre (RS), Brazil.; 7 Complexo Hospitalar da Santa Casa de Porto Alegre Hospital Nora Teixeira Department of Critical Care Porto Alegre RS Brazil Department of Critical Care, Hospital Nora Teixeira, Complexo Hospitalar da Santa Casa de Porto Alegre - Porto Alegre (RS), Brazil.; 8 Brazilian Research in Intensive Care Network São Paulo SP Brazil Brazilian Research in Intensive Care Network (BRICNet) - São Paulo (SP), Brazil.

## INTRODUCTION

A liberalized (i.e., open) visitation policy for family members in intensive care units (ICUs) is endorsed by professional society guidelines as a key element of patient- and family-centered care.^(1)^ Admission to an ICU can evoke a range of emotional responses in both patients and their families, including fear, sadness, anger, and uncertainty.^(2,3)^ When these emotions are compounded by restricted contact between patients and their loved ones, significant psychological distress and increased morbidity are observed.

Although evidence suggests that flexible visitation models are safe and associated with improved satisfaction with care and better short- and long-term mental health outcomes,^(4-11)^ most ICUs continue to impose restrictions on family presence.^(12)^ This gap between evidence and practice may stem from the lack of specific recommendations for implementing liberalized visiting policies, as well as local contextual factors and institutional barriers that hinder the adoption of evidence-based practices.^(13)^ Thus, this viewpoint aims to explore strategies for implementing liberalized family visitation in the ICU.

## LIBERALIZED FAMILY PRESENCE POLICY IMPLEMENTATION

The implementation of a liberalized visitation model in ICUs involves a structured process to ensure success, which hinges on careful planning and execution. This process can be divided into three phases: planning, execution, and evaluation.

### Planning

Implementing a liberalized visitation policy involves creating a model that is tailored to the unique needs of the ICU, its patients, and their families. The planning phase includes forming an implementation team; understanding patient, family, and staff preferences; optimizing the ICU's physical space; and establishing protocols to ensure safety and prevent disruptions to care.

### Implementation team

A multidisciplinary implementation team should oversee the development, execution, and management of the visitation policy, ensuring its integration into the hospital workflow. In addition to health care providers, former patients, family members, and support staff may be included to ensure diverse perspectives.

### Identifying preferences

Surveys can help gather input from patients, families, and ICU staff. Understanding the opinions and impressions of frontline providers — especially nurses — is key to aligning the visitation model with humanized, feasible, and safe care. Social, cultural, and religious factors should also be considered.

### Structure

While the liberalized visitation model does not require extensive changes to the ICU's infrastructure, improvements can enhance comfort and feasibility. Family presence 24/7 has implications for the waiting area and patient rooms.^(14)^ Examples include providing private spaces, toilet and shower facilities, lockers, kitchenettes, and sleep areas in patient rooms.

### Establishing protocols

Clear protocols must be developed to define the roles and responsibilities of family members and other visitors. These protocols should be communicated regularly and may address aspects such as limits on the number of visitors and opportunities for engagement, such as attending multidisciplinary rounds, remaining in the room during invasive bedside procedures, writing ICU diaries, or providing comfort measures such as reading or gentle massage.^(1)^

Open visitation raises legitimate concerns around confidentiality and privacy. Institutions must develop policies regarding the use of electronic devices, visitor conduct, and the protection of patient information. This includes confidentiality agreements, privacy guidelines (physical, visual, and auditory), and staff training on managing potential breaches. Compliance with laws such as the General Data Protection Law or equivalents is essential.

### Visitor education

Educating visitors about the ICU environment, equipment, and visitation policies is essential. This can include orientation sessions, written materials, or apps. Education should address infection prevention, equipment safety, privacy issues, and patient interaction.^(6,9)^

### Psychological and social support

Greater family presence is associated with an increased need for psychological and social support. Emotional strain, anxiety, and acute stress are common experiences for families. Social services can help families navigate socioeconomic barriers, contributing to patient recovery and caregiver resilience. Additionally, providing bereavement support to families of patients who have died in the ICU is an essential component of comprehensive care.

### Staff qualifications

Health care professionals must be trained in communication and empathy to manage increased family interaction. Additionally, staff should receive guidance on privacy, boundaries, and conflict resolution.

### Burnout prevention

Open visitation can increase staff workload, which may contribute to burnout.^(4,15)^ Studies have identified concerns related to emotional labor, time demands, and staff anxiety. Planning should include workload management strategies, such as team-based care models and stress-reduction programs.

### Anticipation of barriers

**Figure 1:**
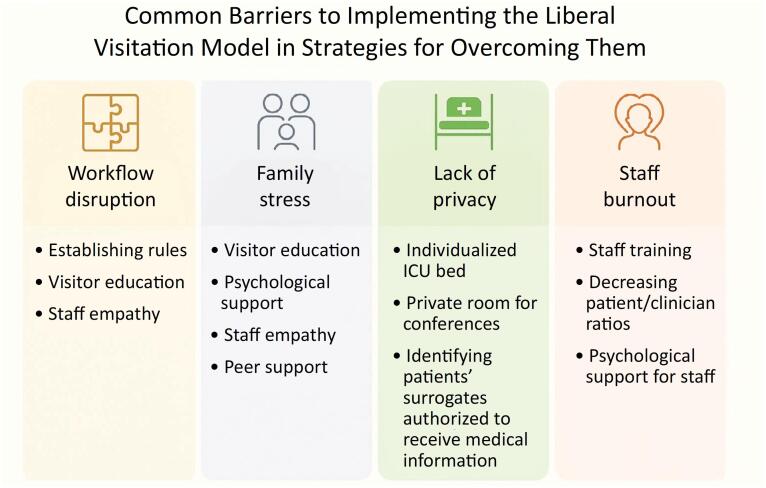
Common barriers to the implementation of liberalized visitation policies in the intensive care unit and recommended strategies.

Identifying potential barriers in the ICU environment, such as disruption of care, lack of privacy, family stress or staff burnout (Figure 1), allows the team to address challenges proactively.^(13)^ For example, logistical issues such as overcrowding or staff resistance can be mitigated through careful planning and communication.

ICU - intensive care unit.

## EXECUTION

Execution involves applying the planned strategies while ensuring flexibility for adjustments. Starting with a pilot program or limited hours can ease the transition toward a full 24/7 visitation model. Tools such as timelines, Plan-Do-Study-Act cycles, root cause analysis, and continuous training should be used.^(13)^ Staff engagement is critical, and leadership should foster motivation through recognition, feedback, and support.

## EVALUATION

Evaluation ensures the effectiveness of a policy, highlighting what works and what needs improvement. Both process (e.g., visit duration, adherence) and outcome indicators (e.g., satisfaction, reported incidents) should be used. Feedback loops should inform iterative improvements. The lessons learned from both successes and setbacks contribute to long-term sustainability.

## KEY CONSIDERATIONS

During implementation, teams must address key questions: Who may visit? What boundaries exist? How is infection prevented? What support exists for family stress? What are staff concerns? Protocols must be implemented. Family participation should be encouraged but never feel obligatory. Importantly, child visitation, while complex, deserves careful attention; we acknowledge the need for separate guidance but briefly note that children may benefit from structured, supported visitation in appropriate cases.

## CONCLUSION

Liberalized visitation in intensive care units represents a shift toward more compassionate, patient- and family-centered care. Despite institutional inertia, the potential benefits — such as increased care quality, reduced patient stress, and stronger patient–family–health care team partnerships — are substantial. This model aligns with contemporary health care values of empathy and shared responsibility. By addressing the logistical, ethical, and emotional dimensions of visitation, intensive care units can create environments that are safer, more humane, and more effective for all.
